# Supplementary figures and images for: HIV-1 Vpr orchestrates ciTRAN upregulation through TGF-β induction
Source: PLoS Pathog. 2025 Jul 9;21(7):e1013332. doi: 10.1371/journal.ppat.1013332 (PMC12266428; doi:10.1371/journal.ppat.1013332)

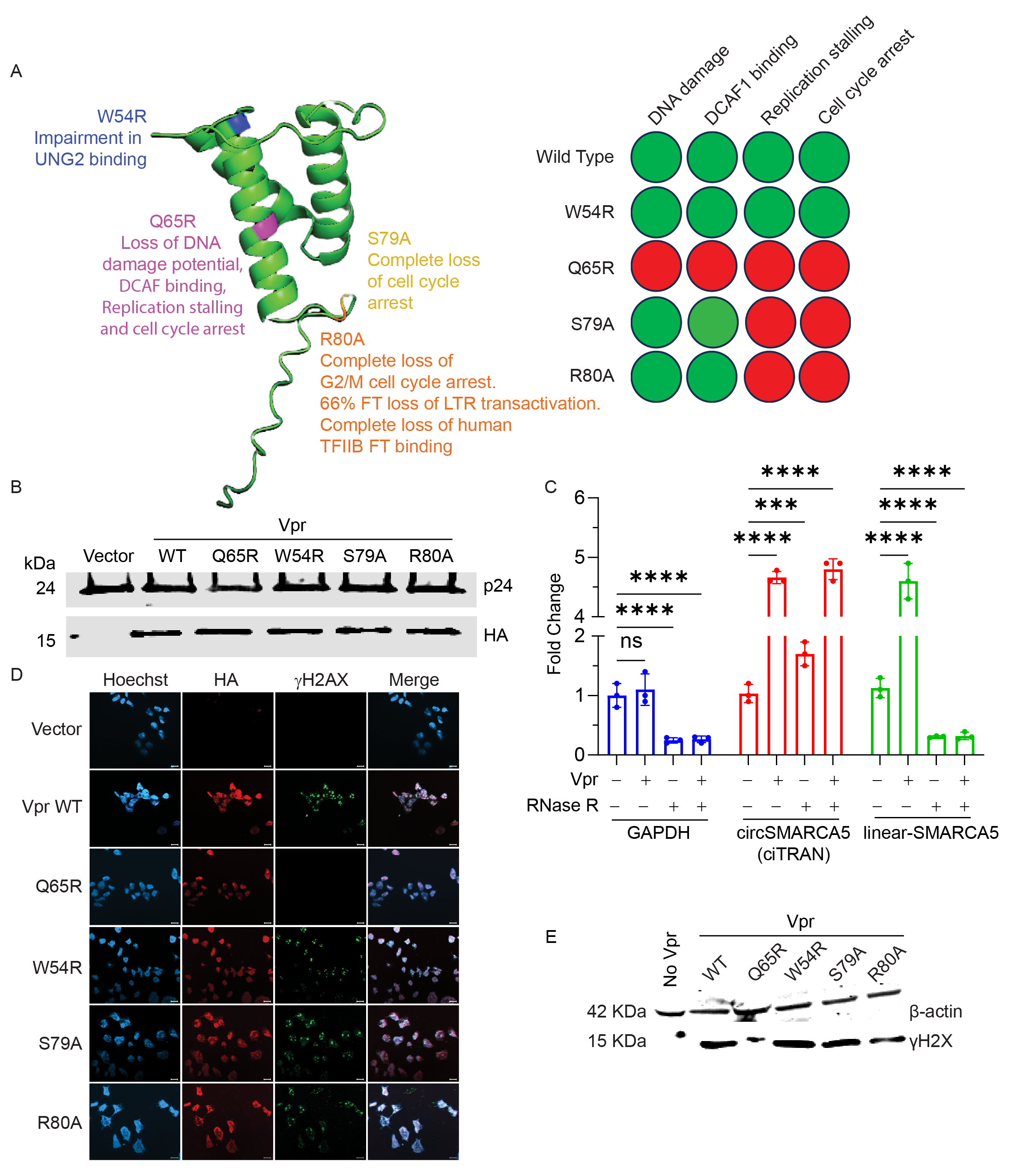

Supplement: S1 Fig — Right panel-summarizing various functions of Vpr mutants: Green indicates proficient and Red indicating deficient in the depicted function. (B) Immunoblot showing packaging of HA-Vpr and its mutants in LVs produced from HEK293T. (C) RNase R treatment (30 mins) was given to RNA isolated from the Vpr or without transduced JTAg cells and levels of circ-SMARCA5, linear SMARCA5, GAPDH was assessed by qRT-PCR. All the data were normalized to control RNaseR samples (n = 3 ± SD). (D) Immunofluorescence of γH2X along with HA-Vpr WT and indicated mutants (Scale bar-100μm). (E) Immunoblot showing the DNA damage marker γH2X with different Vpr mutants with corresponding control as β-actin. (TIF) [file ppat.1013332.s001.tif]

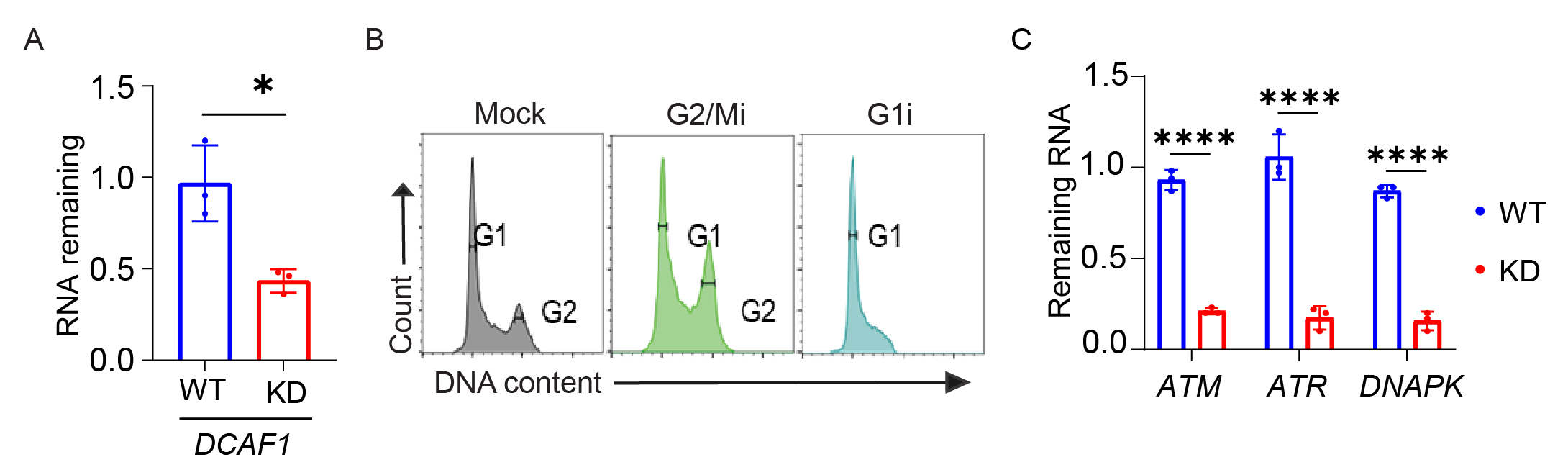

Supplement: S2 Fig — (B) Cell cycle analysis with G2/Mi inhibitor (Apegenin) and G1 inhibitor (CPI 203) and DMSO after 24 hours of treatment in JTAg cells. (C) Knockdown of ATM, ATR, DNAPK in JTAg cells was assessed by qRT-PCR. Data were normalized to GAPDH in each conditions (n = 3 ± SD). (TIF) [file ppat.1013332.s002.tif]

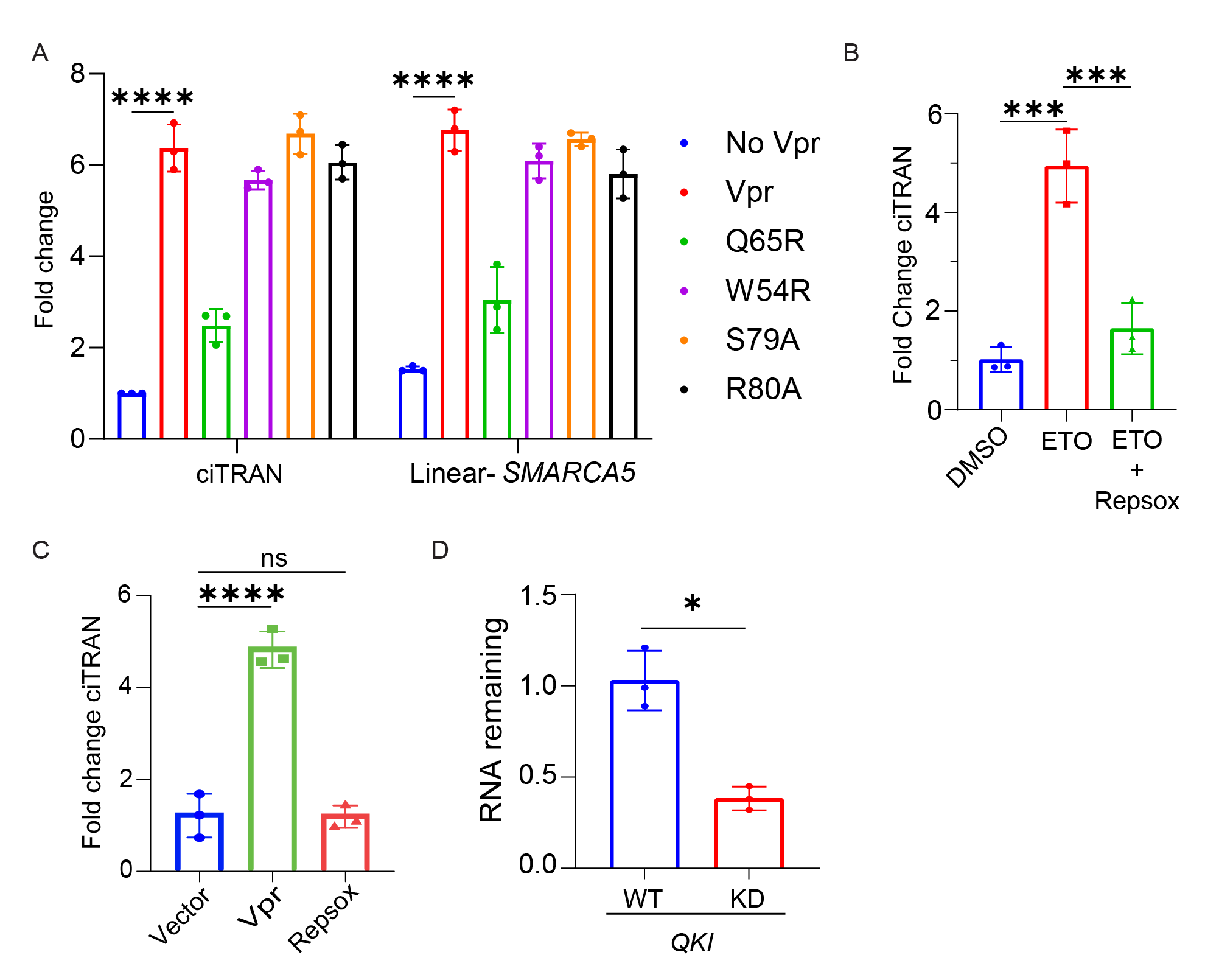

Supplement: S3 Fig — (B) ciTRAN levels were quantified (after 6 h) from CD4+ T cells that received (20µM) of etoposide with or without Repsox (10µM) treatment for 4h. (C) Effect of Repsox on ciTRAN levels was assessed by qRT-PCR from HEK293T cells after 24 hours transfected with either vector or Vpr plasmid or Repsox (10μM) treatment. (D) Levels of QKI post-shRNA-mediated knockdown by qRT-PCR from JTAg cells. Data were normalized to GAPDH. The two-tailed Student’s t-test (unpaired) or one-way ANOVA with Dunnett’s Multiple comparison test was used to assess the significance between two or more groups, ns = non-significant, *p < 0.05, **p < 0.01, ***p < 0.001 and ****p < 0.0001. (TIF) [file ppat.1013332.s003.tif]

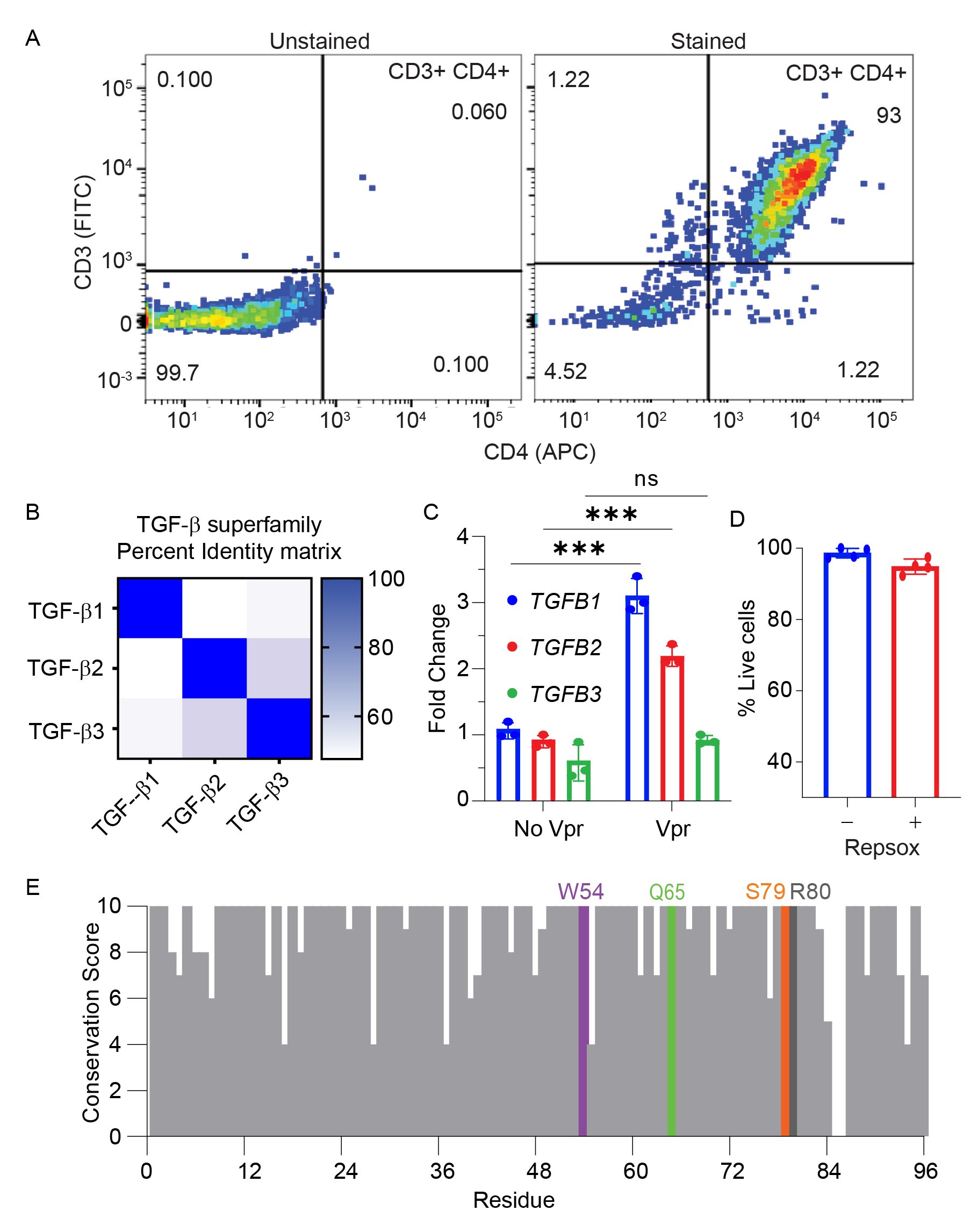

Supplement: S4 Fig — (B) TGF-β family protein comparison from uniport database as, P01137- TGF-β1, P61812- TGF-β2, P10600- TGF-β3 and aligned using clustal omega to determine their percent identity. (C) Analysis of TGFB superfamily members from human primary CD4+ cells by qRT-PCR after transducing with LVs with or without Vpr. (D) Cell viability assay using Alamar blue with/without Repsox in human primary CD4+ cells. (E) Conservation of indicated amino acid residues in Vpr sequences analyzed from the LANL database. (TIF) [file ppat.1013332.s004.tif]

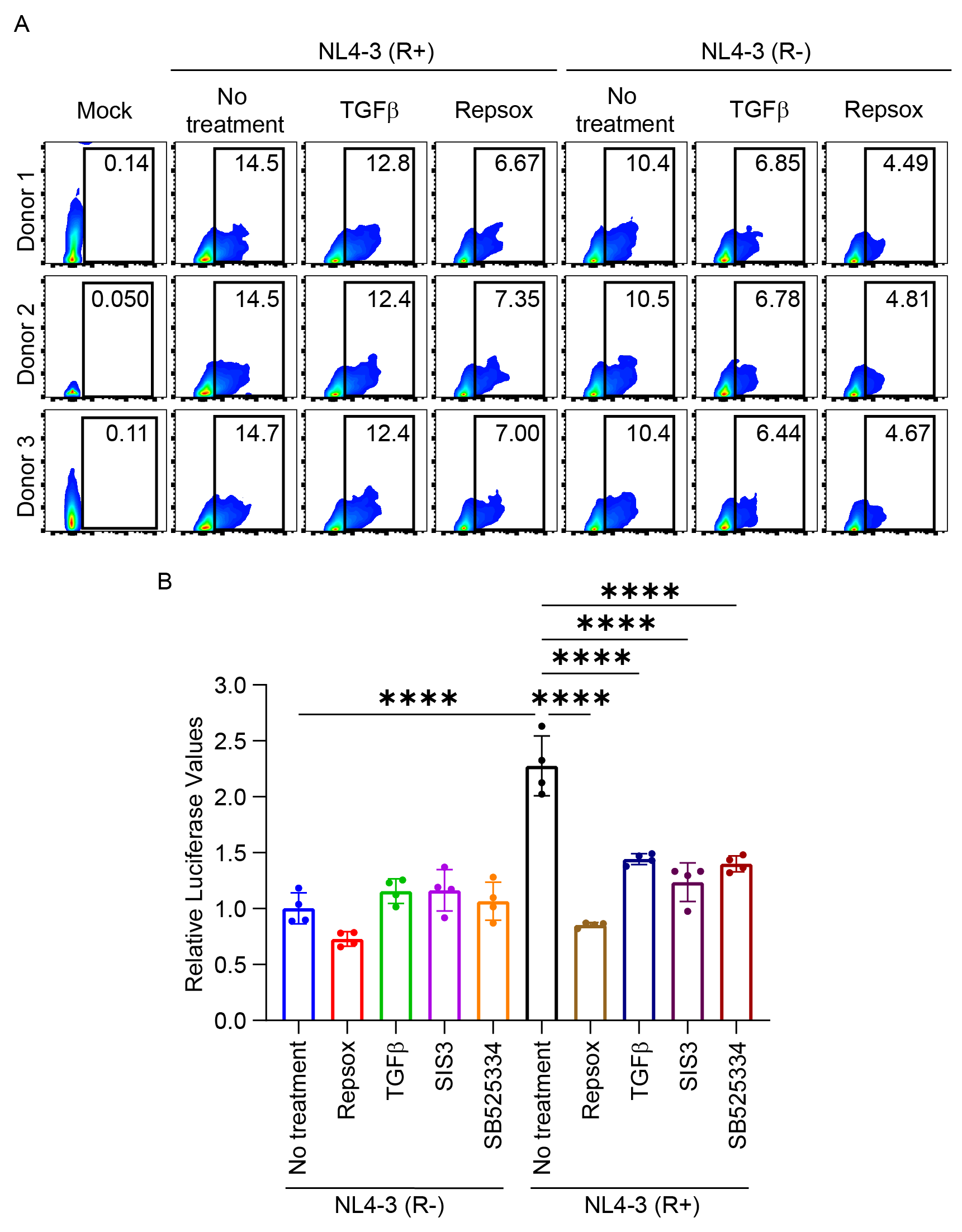

Supplement: S5 Fig — The cells were challenged with HIV-1 NL4–3 (R+) and NL4–3 (R-) viruses and cultured with or without Repsox (10 µM) or TGFβ (10ng/ml). (B) Luciferase activity assay performed after 24 hours following HIV-1 Luc Vpr(+/−) infection of CD4 + primary T cells. presented as biological replicates normalized to total protein quantified by Bradford assay. Two-way ANOVA with Dunnett’s Multiple comparison test was used to assess the significance between two or more groups, ns = non-significant, *p < 0.05, **p < 0.01, ***p < 0.001 and ****p < 0.0001. (TIF) [file ppat.1013332.s005.tif]
