# Supplementary material for: HIV-1 Vpr orchestrates ciTRAN upregulation through TGF-β induction
Source: PLoS Pathog. 2025 Jul 9;21(7):e1013332. doi: 10.1371/journal.ppat.1013332 (PMC12266428; doi:10.1371/journal.ppat.1013332)
Supplement: S1 Table — (PDF) [file ppat.1013332.s006.pdf]

S1 Table A: List of Plasmids used in the study		
1. pcDNA 3.1 BS (-)	(79)	A modified pcDNA derivative with blasticidin selection marker
2. pMD2.G	Addgene#12259	Expression of VSV-G
3. psPAX2	Addgene#12260	2nd generation lentiviral packaging plasmid
4. pScalps zsGreen	Prof. Massimo Pizzato	Lentiviral vector expressing Zsgreen under Cyclophilin A promoter
5. NLBN zsGreen (HIV-1 zsGreen)	(80)	Modified NL4-3 which is devoid of Env, Nef and ZsGreen is placed in place of Nef
6. pCDNA HA	(17)	CMV driven expression of HA
7. pCDNA HA Vpr	(17)	CMV driven expression of HA-tagged Vpr
8. pCDNA HA Vpr Q65R	(17)	CMV driven expression of HA-tagged Vpr mutant Q65R
9. pCDNA HA Vpr W54R	(17)	CMV driven expression of HA- tagged Vpr mutant W54R
10. pCDNA HA Vpr R80A	(17)	CMV driven expression of HA- tagged Vpr mutant R80A
11. pCDNA HA Vpr S79A	(17)	CMV driven expression of HA-tagged Vpr mutant S79A
12. pCDNA HA Vpr H71R	This Paper	CMV driven expression of HA-tagged Vpr mutant S79A
13. pGL3-Basic	(17)	Promoter less vector for measuring the activity of promoter and enhancer sequences with a luciferase assay.
14. pGL3-Basic WT *SMARCA5* Promoter	This Paper	*SMARCA5* Promoter cloned in pGL3 basic vector to check the activity of promoter with luciferase assay
15. pGL3-Basic Mut *SMARCA5* Promoter	This Paper	*Mutant SMARCA5* Promoter cloned in pGL3 basic vector to check the activity of promoter with luciferase assay
16. pLenti CRISPR E TGFβRI gRNA TS1	(59)	For Knockout of TGFβRI

17. pLenti CRISPR E TGFβRI gRNA TS2	(59)	For Knockout of TGFβRI
18. pLKO.1-puro shRNAs	Dharmacon	For Expression of shRNAs.
19. pCH040.c/2625	ARP-11740	Full-Length Transmitted/Founder (T/F) HIV-1 Infectious Molecular Clones
20. pWITO.c/2474	ARP-11739	Full-Length Transmitted/Founder (T/F) HIV-1 Infectious Molecular Clones
21. pSUMA.c/2821	ARP-11748	Full-Length Transmitted/Founder (T/F) HIV-1 Infectious Molecular Clones
22. pRHPA.c/2635	ARP-11744	Full-Length Transmitted/Founder (T/F) HIV-1 Infectious Molecular Clones
23. pTRJO.c/2851	ARP-11747	Full-Length Transmitted/Founder (T/F) HIV-1 Infectious Molecular Clones
24. pREJO.c/2864	ARP-11746	Full-Length Transmitted/Founder (T/F) HIV-1 Infectious Molecular Clones
25. pTHRO.c/2626	ARP-11745	Full-Length Transmitted/Founder (T/F) HIV-1 Infectious Molecular Clones
26. SBE4-Luc	Addgene#1 6495	SMAD reporter plasmid
27. pGL3 NL4-3 LTR	(17)	LTR-Luciferase Reporter
28. pGL3 RHPA LTR	(17)	LTR-Luciferase Reporter
29. pGL3 SUMA LTR	(17)	LTR-Luciferase Reporter
30. pGL3 TRJO LTR	(17)	LTR-Luciferase Reporter
31. pGL3 WITO LTR	(17)	LTR-Luciferase Reporter
32. pcDNA HIV-1 Tat	(80)	Expression of HIV-1 Tat from CMV promoter.
33. pLenti CRISPR E CXCR4 gRNA	(59)	For Knockout of CXCR4
34. pNL4-3 Env- R- Luc	(22,87)	Envelope, Nef and Vpr mutant and Luc is placed in place of Nef
35. pNL4-3 Env- R+ Luc	(22,87)	Envelope and Nef mutant and Luc is placed in place of Nef
36. pNL4-3 Env+ R+ Luc	(88)	For replication Kinetics study
37. pNL4-3 Env+ R- Luc	This study	For replication Kinetics study Vpr mutant

S1 Table B: List of Reagents used in the study		
Reagent	Company	Catalog No.
1. Dulbecco's Modified Eagle Medium (DMEM)	Gibco, USA	12100046
2. RPMI 1640	Gibco, USA	11875093
3. Advanced DMEM	Gibco, USA	12491-015
4. Advanced RPMI	Gibco, USA	12633-012
5. Fetal Bovine Serum (FBS), Certified, Performance tested. Origin: US	Gibco, USA	10082-147
6. Fetal Bovine Serum (FBS), Collected in South America	Capricorn Scientific	FBS12-A
7. HiFi Peripheral Blood Mononuclear cells (H-PBMC), Pooled three donors, CL10-10	HiMedia	Lot No.0000522977
8. HiFi Peripheral Blood Mononuclear cells (H-PBMC), Single donor,CL1003-10	HiMedia	Lot No.0000654440
9. HiFi Peripheral Blood Mononuclear cells (H-PBMC), Single donor, CL1003-10	HiMedia	Lot No.0000651792
10. HiFi Peripheral Blood Mononuclear cells (H-PBMC), Single donor, CL1003-10	HiMedia	Lot No. 0000653326
11. L-Glutamax supplement	Gibco, USA	35050061
12. Trypsin	Gibco	25200-056
13. Hoechst 33342	Sigma Aldrich	14540
14. Opti-MEM I	Gibco, USA	22600-050
15. PBS	HyClone, USA	SH30256.02
16. Paraformaldehyde	Sigma Aldrich	F1635-25ML
17. Tris(2-carboxyethyl) phosphine hydrochloride (TCEP)	Sigma Aldrich	75259
18. 2-Mercaptoethanol	Sigma	M6250
19. cOmplete, EDTA-free Protease Inhibitor Cocktail	Roche	04693132001
20. Sodium Orthovandate	Sigma	S6508
21. Sodium fluoride	Sigma	201154
22. n-Dodecyl-β-D-Maltoside	Thermo Fischer	89903
23. HEPES	VWR Life Science	051-250G
24. Sodium Chloride	Promega	H5273
25. PVDF membrane	Immobilon-FL, Merck-Millipore	IPFL00010 R7DA8781C
26. Tricine	MP Biomedicals	103112 QR15009

27. Biorad Blocking Buffer	Bio rad	12010020
28. Odyssey Blocking Buffer (TBS)	LI-COR Biosciences	927-50000
29. Lipofectamine 3000 Transfection Reagent	Thermo Fisher Scientific	L3000008
30. Tween-20	Sigma Aldrich	P2287-500ML
31. BD Perm/Wash Perm/Wash Buffer	BD Biosciences	554723
32. Luciferin	Cayman Chemicals	14682-50MG
33. Dynabeads Protein G	Invitrogen	10003D
34. SYBR Green-I	Thermo Fisher Scientific	S-7585
35. Maxima SYBR Green/Fluorescein qPCR Master Mix	Thermo Scientific	K0241
36. TRI Reagent	Sigma	T9424
37. QIAzol Lysis Reagent	QIAGEN	79306
38. TRIzol Reagent	Invitrogen	15596018
39. Revert Aid H minus RT	Thermo Fisher Scientific	K1632
40. RNAse R	Lucigen	RNR07250
41. α-Amanitin	Sigma Aldrich	B2263
42. Etoposide	Tocriscreen Plus Micro	6455
43. Doxorubicin	Tocriscreen Plus Micro	6455
44. Recombinant TGF-β	Gibco	PHG9214
45. Propidium Iodide	Sigma	537059
46. AZ 20	Tocriscreen Plus Micro	6455
47. SB 525334	Tocriscreen Plus Micro	6455
48. Apigenin	LOPAC®1280	LO3300
49. CPI 203	Sigma	6455
50. Repsox	Sigma	R0158-5MG
51. DMSO	Cayman Chemical Company	D8418
52. KU-55933	Cayman Chemical Company	16336
53. NU 7441	Cayman Chemical Company	14881
54. Gibco Human IL-2 Recombinant ProteinL-2	Gibco	PHC0026
55. Purified anti-HA.11 Epitope Tag antibody	BioLegend	901501
56. Beta-Actin Rabbit Monoclonal Antibody	LI-COR Biosciences	926-42210
57. Beta-Actin Mouse Monoclonal Antibody	LI-COR Biosciences	926-42212
58. beta Actin Loading Control Monoclonal Antibody (BA3R), DyLight 680	Thermo Fisher Scientific	MA5-15739-D680
59. IRDye 680RD Goat anti-Mouse IgG antibody	LI-COR Biosciences	925-68070

60. IRDye 800CW Goat anti-Rabbit IgG antibody	LI-COR Biosciences	925-32211
61. IRDYe 680 Donkey anti-Goat IgG antibody	Thermo Fisher Scientific	NC0272752
62. Anti-HIV-1 p24 Hybridoma	NIH-ARP	1513
63. Anti- HIV-1 HXB2 Tat Monoclonal	NIH-ARP	7377
64. Rpb1 CTD mouse Antibody	Cell signalling Technology	2629
65. Human anti-CD4-APC	Miltenyi Biotec	130-113-812
66. Human anti-CD3-FITC	Miltenyi Biotec	130-113-690
67. Goat TGFβ RI	R&D Systems	AF3025
68. Rabbit SMAD2/3	CST	77397
69. Rabbit Smad2	CST	77397
70. Mouse anti-SRSF1	Thermo Fisher Scientific	324500
71. Mouse anti-BrdU	Sigma-Aldrich	B8434
72. Purified anti-H2A.X Phospho (Ser139) Antibody	Biolegend	613401
73. Anti HA rabbit monoclonal antibody(C29F4)	CST	3724S
74. Phospho-SMAD2/SMAD3	Thermo Fisher Scientific	MA5-51556
75. RNA Clean and Concentrator-5	Zymo Research	R1014
76. Nucleospin Gel and PCR clean-up kit	Macherey-Nagel	740609
77. NucleoSpin Plasmid Transfection-grade, Mini kit for ultrapure plasmid DNA	Machery-Nagel	740490
78. NucleoBond Xtra Midi EF, Midi kit for endotoxin-free plasmid DNA	Machery-Nagel	740420
79. NucleoSpin Tissue, Mini kit for DNA from cells and tissue	Machery-Nagel	740952
80. Biolegend ELISA TGF-β1 Kit	Biolegend	436707
81. CD4+ T Cell Isolation Kit, human	Miltenyi Biotec	130-045-101
82. Nuclease-free water	Thermo Fisher Scientific	AM9937

**S1 Table C: List of Software used in the study**		
1. GraphPad Prism	GraphPad Software	https://graphpad.com
2. ImageJ		
