# Supplementary material for: HIV-1 Vpr orchestrates ciTRAN upregulation through TGF-β induction
Source: PLoS Pathog. 2025 Jul 9;21(7):e1013332. doi: 10.1371/journal.ppat.1013332 (PMC12266428; doi:10.1371/journal.ppat.1013332)

Data Table S1: oligos sequences

Gene	Sequences	
HIV1 Gag-Forward	TTGTACTGAGAGACAGGCT	For HIV-1 transcription quantification
HIV1 Gag-Reverse	ACCTGAAGCTCTCTTCTGG	
QKI-Forward	AAGCCCACCCCAGATTACCT	QKI RT-qPCR primers
QKI-Reverse	ACTCTGCTAATTTCTTCGTCCAG	
SMARCA 5 Promoter-Forward	ATATGGTACCGATTGCAGTGAGCTGAGATC	*SMARCA* 5 Promoter cloning in pGL3-Basic
SMARCA 5 Promoter-Reverse	TGGCGGCACTACCGGAAAACT	
SMARCA 5 Promoter Mutation	GTTTTGGGGCTATAACACAAGACTTC	*SMARCA* 5 Promoter Mutation
VPR-Forward	GACTACAAGGACGACGATGACAAGATGGAACAAGCC	VPR Cloning
VPR-Reverse	GGGCTCTAGTCTAGGATCTAC	
Q65R	AAGAATTCTGAGACAACTGCTGTTTA	VPR Mutation
W54R	ACGGGGATACTAGAGCAGGAGTGGAAGCC	
S79A	GGGTGTCGACATGCCAGAATAGGCGTT	
R80A	TGTCGACATAGCGCCATAGGCGTTACT	
H71R	TGCTGTTTATCCGATTCAGAATTGG	VPR Mutation
SERPINE1-Forward	GCAGGACATCCGGGAGAGA	SMAD2 ChIP positive control
SERPINE1-Reverse	CCAATAGCCTTGGCCTGAGA	
HPRT1-Forward	TGTTTGGGCTATTTACTAGTTG	SMAD2 ChIP negative control
HPRT1-Reverse	ATAAAATGACTTAAGCCCAGAG	
SMARCA 5 Promoter-Forward	GTGGCGTCACGCCTCTAAGCGG	*SMARCA* 5 Promoter RT-qPCR Primer
SMARCA 5 Promoter-Reverse	TGGCGGCACTACCGGAAAACT	
ATM-Forward	TGTTCCAGGACACGAAGGGAGA	ATM RT-qPCR primers
ATM-Reverse	CAGGGTTCTCAGCACTATGGGA	
ATR-Forward	GGAGATTTCCTGAGCATGTTCGG	ATR RT-qPCR primers
ATR-Reverse	GGCTTCTTTACTCCAGACCAATC	
DNA-PK -Forward	GAGAAGGCGGCTTACCTGAG	DNA-PK RT-qPCR primers
DNA-PK -Reverse	CGAAGGCCCGCTTTAAGAGA	
TGFB1-Forward	TCGCCAGAGTGGTTATCTT	TGFB1 RT-qPCR primers
TGFB1-Reverse	TAGTGAACCCGTTGATGTCC	
TGFB1-Forward	GGACATCAACGGGTTCACTAC	
TGFB1-Reverse	TAGTTGGTGTCCAGGGCTCG	
TGFB2-Forward	ACACTCAGCACAGCAGGGTCCT	TGFB2 RT-qPCR primers
TGFB2-Reverse	TTGGGACACGCAGCAAGGAGAAG	
TGFB3-Forward	TGAGTGGCTGTTGAGAAGAGA	TGFB3 RT-qPCR primers
TGFB3-Reverse	ATTGTCCACGCCTTTGAATTTGAT	
ciTRAN-Forward	TCTCCAAGATGGGCGAAAGT	ciTRAN RT-qPCR primers
ciTRAN-Reverse	ACATGTGTTGCTCCATGTCT	
SMARCA 5-Forward	ATTGCATTCACAGAGTGGATT	*SMARCA* 5 RT-qPCR primers
SMARCA 5-Reverse	AGTTTTTCTGTAAAACAGAATTT	
DCAF1-Forward	TGAGGGTGGCATTCTTGTCC	DCAF1 RT-qPCR primers
DCAF1-Reverse	TCCCAATATAGCTGCGCTGG	
β-actin-Forward	GACAGGATGCAGAAGGAGATTACTG	β-actin RT-qPCR primers
β-actin-Reverse	CTCAGGAGGAGCAATGATCTTGAT	
GAPDH-Forward	AACAGCGACACCCACTCCTC	GAPDH RT-qPCR primers
GAPDH-Reverse	CATACCAGGAAATGAGCTTGACAA	

shRNA sequences	
QKI	CCGGGAAGCAGAAACCGGATGTAAACTCGAGTTTACATCCGGTTTCTGCTTCTTTTTG
QKI	CCGGCCGAAGCTGGTTTAATCTATACTCGAGTATAGATTAAACCAGCTTCGGTTTTTG
SMAD2	CCGGCCAGTAATAGTTGCATTGATACTCGAGTATCAATGCAACTATTACTGGTTTTTG
SMAD2	CCGGCCCATCAAATTCAGAGAGGTTCTCGAGAACCTCTCTGAATTTGATGGGTTTTTG
SMAD2	CCGGGCGTTGCTCAAGCATGTCATACTCGAGTATGACATGCTTGAGCAACGCTTTTTG
SMAD2	CCGGCGATTAGATGAGCTTGAGAAACTCGAGTTTCTCAAGCTCATCTAATCGTTTTTG
SMAD2	CCGGCCTAAGTGATAGTGCAATCTTCTCGAGAAGATTGCACTATCACTTAGGTTTTTG
ATM	CCGGCTGGTGACTATACAGTCATTTCTCGAGAAATGACTGTATAGTCACCAGTTTTTTG
ATM	CCGGCAAACGAAATCTCAGTGATATCTCGAGATATCACTGAGATTTCGTTTGTTTTTTG
ATM	CCGGCCTACTTTGTGCAGGTCATACCTCGAGGTATGACCTGCACAAAGTAGGTTTTTTG
ATM	CCGGGTATTACCTTTCGTGGTATAACTCGAGTTATACCACGAAAGGTAATACTTTTTTG
ATR	CCGGCTGTGGTTGTATCTGTTCAATCTCGAGATTGAACAGATACAACCACAGTTTTTG
ATR	CCGGCCGGATACTTACAGATGTAAACTCGAGTTTACATCTGTAAGTATCCGGTTTTTG
ATR	CCGGGCCGCTAATCTTCTAACATTACTCGAGTAATGTTAGAAGATTAGCGGCTTTTTG
ATR	CCGGGCCAAAGTATTTCTAGCCTATCTCGAGATAGGCTAGAAATACTTTGGCTTTTTG
ATR	CCGGGCTGATTATTTACAACCCAAACTCGAGTTTGGGTTGTAAATAATCAGCTTTTTG
DNAPK	CCGGGCAGATAGAAAGCATTACATTCTCGAGAATGTAATGCTTTCTATCTGCTTTTT
DNAPK	CCGGCCGGTAAAGATCCTAATTCTACTCGAGTAGAATTAGGATCTTTACCGGTTTTT
DNAPK	CCGGCCAGTGAAAGTCTGAATCATTCTCGAGAATGATTCAGACTTTCACTGGTTTTT
DNAPK	CCGGCCACCCTTTGTCTCTTGTATTCTCGAGAATACAAGAGACAAAGGGTGGTTTTT
DNAPK	CCGGGCAGCCTTATTACAAAGACATCTCGAGATGTCTTTGTAATAAGGCTGCTTTTT
DCAF1	CCGGCGAGAAACTGAGTCAAATGAACTCGAGTTCATTTGACTCAGTTTCTCGTTTTTTG
DCAF1	CCGGGCGCCAATAAACTTTACGTCACTCGAGTGACGTAAAGTTTATTGGCGCTTTTTTG
DCAF1	CCGGCCTCCCATTCTTCTGCCTTTACTCGAGTAAAGGCAGAAGAATGGGAGGTTTTTTG
DCAF1	CCGGGCTGAGAATACTCTTCAAGAACTCGAGTTCTTGAAGAGTATTCTCAGCTTTTTTG
DCAF1	CCGGCGTATCGCTAATGGCATTGCACTCGAGTGCAATGCCATTAGCGATACGTTTTTTG

gRNA sequences	
TGFβRI gRNA	CATACAAACGGCCTATCTCG
TGFβRI gRNA	ATTGCTCGACGATGTTCCAT
CXCR4 gRNA	GTTCCAGTTTCAGCACATCA

Related to Fig 1B

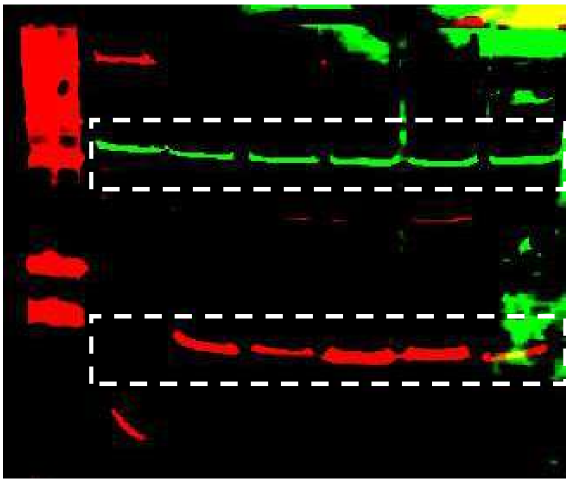

Related to Fig 2B

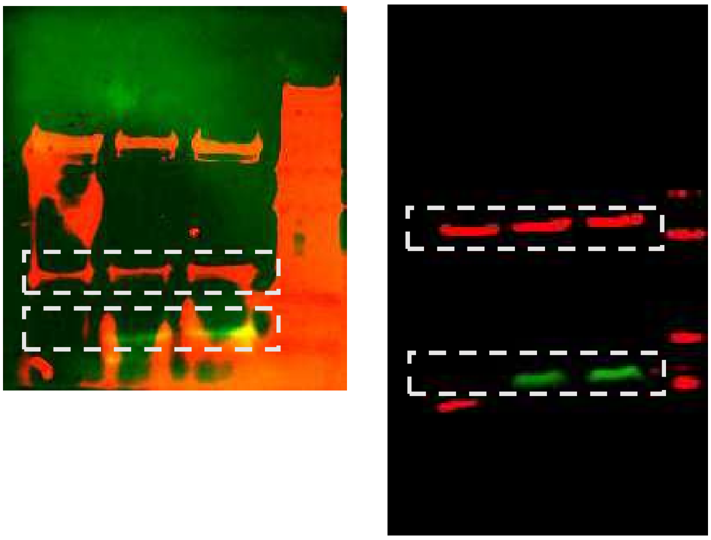

Related to Fig 3D

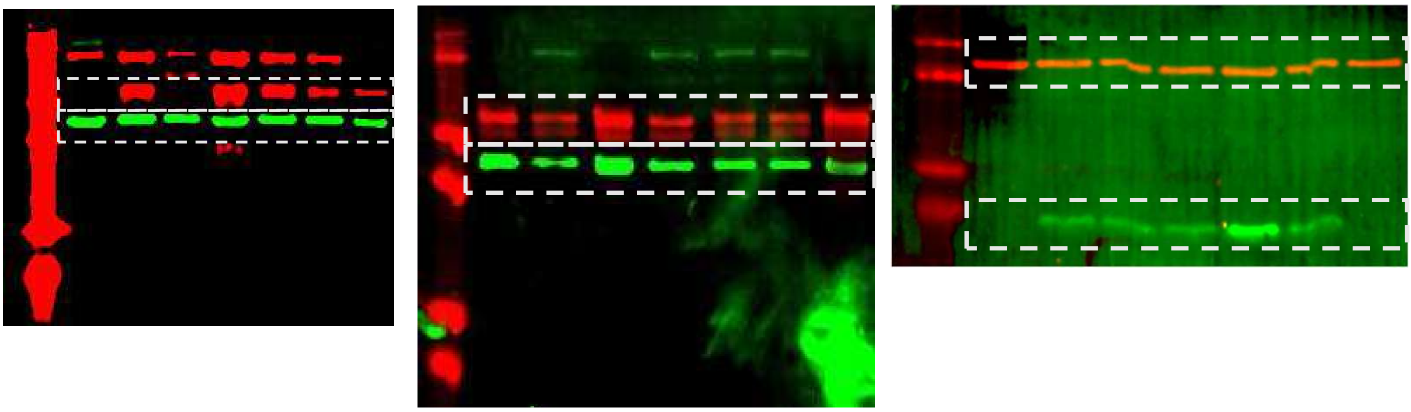

Related to Fig 3G

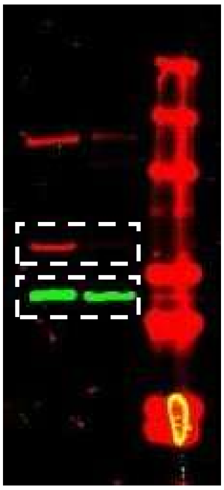

Related to Fig 3H

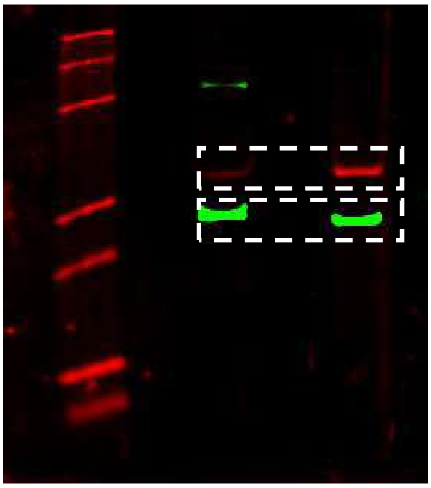

Related to Fig S1B

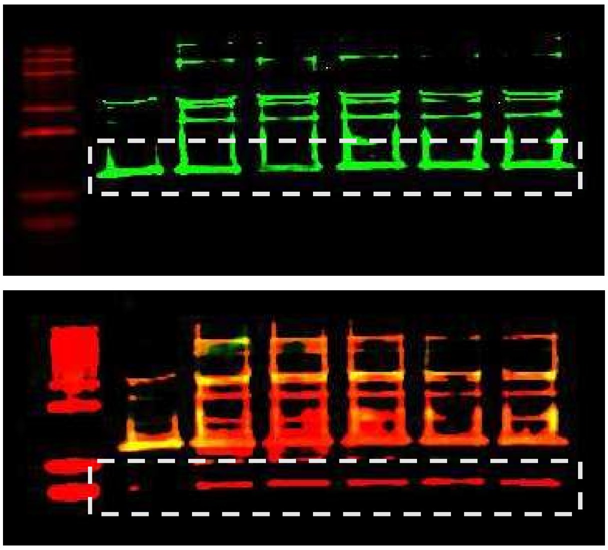

Related to Fig S1E

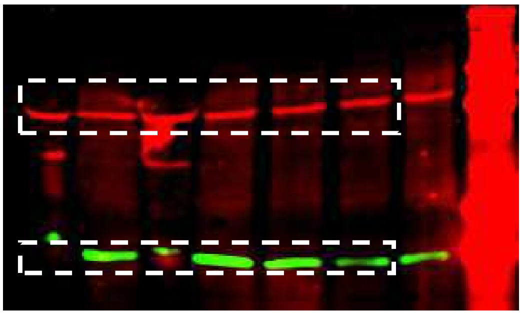

Supplement: S1 File — (PDF) [file ppat.1013332.s007.pdf]
